# Tumor-restrictive type III collagen in the breast cancer microenvironment: prognostic and therapeutic implications

**DOI:** 10.21203/rs.3.rs-2631314/v1

**Published:** 2023-04-12

**Authors:** Becky K. Brisson, Bassil Dekky, Ashton C. Berger, Elizabeth A. Mauldin, Claudia Loebel, William Yen, Daniel C. Stewart, Deborah Gillette, Charles-Antoine Assenmacher, Edna Cukierman, Jason A. Burdick, Virginia F. Borges, Susan W. Volk

**Affiliations:** 1 Department of Clinical Sciences and Advanced Medicine, School of Veterinary Medicine, University of Pennsylvania, Philadelphia, Pennsylvania, USA; 2 Department of Pathobiology, School of Veterinary Medicine, University of Pennsylvania, Philadelphia, Pennsylvania, USA; 3 Department of Bioengineering, School of Engineering and Applied Science, University of Pennsylvania, Philadelphia, Pennsylvania, USA; 4 Department of Materials Science & Engineering, University of Michigan, Ann Arbor, Michigan, USA; 5 Cancer Signaling and Microenvironment Program, The Martin and Concetta Greenberg Pancreatic Cancer Institute, Fox Chase Cancer Center, Temple University Lewis Katz School of Medicine, Philadelphia, Pennsylvania, USA; 6 BioFrontiers Institute and Department of Chemical and Biological Engineering, University of Colorado Boulder, Boulder, Colorado, USA; 7 Department of Medicine, Division of Medical Oncology, University of Colorado Anschutz Medical Campus, Aurora, Colorado, USA; 8 University of Colorado Cancer Center, Aurora, Colorado, USA; 9 Young Women’s Breast Cancer Translational Program, University of Colorado Anschutz Medical Campus, Aurora, Colorado, USA

**Keywords:** tumor microenvironment, type III collagen, mammary tumors, breast cancer, metastasis, collagen, TNBC, BRCA, tumor stroma

## Abstract

Collagen plays a critical role in regulating breast cancer progression and therapeutic resistance. An improved understanding of both the features and drivers of tumor-permissive and -restrictive collagen matrices are critical to improve prognostication and develop more effective therapeutic strategies. In this study, using a combination of *in vitro, in vivo and in silico* experiments, we show that type III collagen (Col3) plays a tumor-restrictive role in human breast cancer. We demonstrate that Col3-deficient, human fibroblasts produce tumor-permissive collagen matrices that drive cell proliferation and suppress apoptosis in noninvasive and invasive breast cancer cell lines. In human TNBC biopsy samples, we demonstrate elevated deposition of Col3 relative to type I collagen (Col1) in noninvasive compared to invasive regions. Similarly, *in silico* analyses of over 1000 breast cancer patient biopsies from The Cancer Genome Atlas BRCA cohort revealed that patients with higher Col3:Col1 bulk tumor expression had improved overall, disease-free and progression-free survival relative to those with higher Col1:Col3 expression. Using an established 3D culture model, we show that Col3 increases spheroid formation and induces formation of lumen-like structures that resemble non-neoplastic mammary acini. Finally, our *in vivo* study shows co-injection of murine breast cancer cells (4T1) with rhCol3-supplemented hydrogels limits tumor growth and decreases pulmonary metastatic burden compared to controls. Taken together, these data collectively support a tumor-suppressive role for Col3 in human breast cancer and suggest that strategies that increase Col3 may provide a safe and effective modality to limit recurrence in breast cancer patients.

## INTRODUCTION

Despite advances in prevention, diagnostics and clinical care of breast cancer patients, breast cancer is currently the most common cancer in women and is a leading cause of cancer-related death worldwide^1–3^. Biologic behavior of breast cancer cells is directed in part by their highly complex but influential microenvironment, consisting of inflammatory cells, cancer-associated fibroblasts (CAFs), endothelial cells, cytokines, growth factors, and the extracellular matrix (ECM). Understanding mechanisms by which the interactions between cancer cells and their microenvironment direct tumor growth and progression, and identification of drivers of the tumor-permissive niche are needed in order to improve clinical outcomes in breast cancer patients.

Collagen is the most abundant protein in the ECM of breast tumors^4^. Increased stromal collagen content and alignment correlates with mammographic density and is one of the strongest predictors of breast cancer risk in women^5^. However, many recent studies have clarified both tumor-restrictive and tumor-permissive roles that collagens play during the development and progression of cancer^6–9^. Alterations in collagen type, density, architecture, and ECM stiffness provide physical, biochemical, and biomechanical cues to neoplastic and stromal cells, influencing their differentiation, proliferation, migration, and invasion^10–18^. Mechanisms by which collagens regulate cell activities and fate in solid tumors and their metastatic niches have focused primarily on type I collagen (Col1). To date, numerous studies have shown that Col1 is increased in multiple types of cancer and associates with aggressive tumor behaviors such as increased proliferation and resistance to apoptosis^19–24^.

In addition to type I collagen, other collagens such as types II, III, V, VI, IX, X and XI are found to be increased in the tumor microenvironment (TME)^25–31^. Type III collagen (Col3) is the second most abundant collagen in tissues and is often coexpressed with Col1. While Col3 is known to play a critical function in tissue and organ maintenance, in part through its ability to regulate Col1 fibrillogenesis^32^, our recent studies have defined a clearly distinct role of Col3 in tissue maintenance, repair and cancer^33–37^. In fact, our data were the first to show that Col3 plays a tumor-suppressive role in the breast cancer microenvironment through direct effects on cancer cells and importantly those mediated by CAF/ECM functional unit-induced collagen microarchitecture^37^. However, whether this information about the levels of Col3 in the TME could be applied prognostically or therapeutically to potentially improve breast cancer care, remains unknown and constitutes the focus of this study.

Expression data in breast cancer biopsy samples from human patients has failed to clearly show such a tumor-suppressive role for Col3 in clinical outcome. While some have suggested that Col3 is a critical component of a distinct stromal response associated with improved clinical outcome^38^, other studies have suggested pro-tumorigenic roles of Col3 by demonstrating its increased expression in the TME of cancers including head and neck squamous cell, breast, pancreas, and colorectal cancer^20,28,39–44^. We hypothesized that an underappreciation of the heterogeneity of the TME^45,46^ as well as recognition of the desmoplastic response may contribute to discrepancies in the literature regarding a prognostic role for Col3 in breast cancer patients from previous genomic and proteomic studies. To address this issue, we interrogated data from 1,099 TCGA BRCA patient tumors using an *in silico* approach that stratified relative Col1 and Col3 expression levels to determine if a higher relative Col3 expression associates with a less aggressive clinical course. Additionally, given that our previous work^37^ and other aligning findings^38,47,48^ support a tumor restrictive role for Col3 in breast cancer, we tested the hypothesis proposed in our initial study that enrichment or delivery of Col3 may control aggressive breast cancer phenotypes.

In this study, we sought to develop a deeper understanding of the role of Col3 in regulating cell behaviors and fate in human breast cancer and advance our understanding of Col3 biology for prognostic and therapeutic applications. By using a combination of *in vitro, in vivo* and *in silico* analyses, we have defined a tumor-suppressive regulatory role for Col3 on human fibroblastic/ECM functional units and concomitant breast cancer cell phenotypes, elucidated prognostic potential of Col3 expression in breast cancer biopsy samples and revealed the impact of Col3-containing hydrogels on tumor growth and metastasis. Collectively, these data support that stromal Col3 plays a tumor suppressive role in human breast cancer and provides proof-of-concept that Col3-based (e.g., tissue engineering) strategies may provide a safe and effective approach to limit recurrence in breast cancer patients.

## MATERIAL AND METHODS

### Ethical Collection of Human Samples

Twenty-three breast cancer samples were obtained from the Young Women’s Breast Cancer cohort tissue repository and database (University of Colorado School of Medicine, Aurora, CO USA; IRB # 09-0853 and #05-0958). Institutional Review Board (IRB) approval for collection and analysis of human tissues was obtained from the Colorado Multi-institutional Review Board (COMIRB). Samples were previously collected prospectively after written patient informed consent or retrospectively via a consent waiver approved protocol. TNBC biopsy samples with associated DCIS and invasive components were selected for inclusion for this study and provided as deidentified specimens. These deidentified samples were determined to not meet the definition of “human subject” by the Institutional Review Board at the University of Pennsylvania.

### BALB/c wild-type and Col3-deficient mice

Animal utilization and care were approved by the Institutional Animal Care and Use Committee of the University of Pennsylvania and followed guidelines set forth in the NIH Guide for the Care and Use of Laboratory Animals. Mice for this study (wild-type (Col3^+/+^), Col3 heterozygous (Col3^+/−^), and Col3 homozygous knockout (Col3^−/−^)) were bred in a colony established at the University of Pennsylvania^37^. Mice were housed in a 12-hour light/dark cycle in a specific pathogen-free facility with controlled temperature and humidity and were provided with food and water ad libitum. Mice were microchipped and microchip numbers were used to identify mice and subsequent samples to maintain blinding with respect to genotype or treatment group throughout experiments.

### Cell culture

Human skin fibroblasts (Detroit 551-CCL-110), human breast cancer cell lines (MCF-7, MDA-MB-453 and MDA-MB-231), and mouse mammary cancer cell line (4T1) were obtained from the American Type Culture Collection (ATCC; Manassas, VA, USA). All cell lines were authenticated by morphology, growth characteristics, and biological behavior, and were routinely tested for Mycoplasma. All cells were used within 7 passages after arrival. Cancer cells were maintained in complete medium: Dulbecco’s modified Eagle’s medium (Glutamax; Gibco, Grand Island, NY) supplemented with 10% fetal bovine serum (Atlanta Biologicals, Flowery Branch, GA) and antibiotics (100 U/mL penicillin and 100 g/mL streptomycin). Human skin fibroblasts were maintained in complete medium: Eagle’s Minimum Essential Medium (ATCC) with the addition of L-ascorbic acid (0.1mg/ml, A8960; Sigma-Aldrich) and 10% fetal bovine and antibiotics as listed above. Cells were routinely incubated at 37°C and 5% CO_2_ under a humidified atmosphere.

### siRNA knock-down of Col3

Human fibroblasts were plated at 1.1-1.8x10^4^ cells/cm^2^ in cell culture dishes. Human type III collagen siRNA (sc-43062), and control siRNA (sc-37007) were purchased from Santa Cruz Biotechnology (Santa Cruz, CA). Transfection was performed using Lipofectamine RNAimax (Invitrogen^®^, Life Technologies) and 10-15nM siRNA according to manufacturer instructions.

### Generation of Fibroblast-Derived Matrices (FDMs) from Fibroblastic/ECM Functional Units.

Decellularized FDMs were obtained as previously described^37,47,49,50^, with the modification that siRNA control-treated (siCtrl) and the siRNA Col3-treated (siCol3) fibroblasts were seeded on 7 or 14 mm glass diameter microwell 35 mm dishes for the generation of the fibroblastic/ECM functional units (MatTek Corporation, Ashland, MA). After 96 hours of siRNA transfection, the functional units were decellularized to render FDMs and stored in PBS with antibiotics at 4°C until used.

### RNA isolation and gene expression analysis by quantitative real-time PCR

Total RNA was isolated from siRNA-treated fibroblasts or frozen tissue samples using RNeasy Mini kits (Qiagen, Venlo, Netherlands). RNA concentration was quantified, and 1μg was subjected to single-strand reverse transcription using the Superscript III First-Strand Synthesis System (Life Technologies) according to the manufacturer’s protocol. The resultant cDNA was used for real-time PCR with oligonucleotides that were specific for *COL3A1* (Fwd: 5’-CTGGACCCCAGGGTCTTC-3’; Rev: 5’-CATCTGATCCAGGGTTTCCA-3’), *COL1A1* (Fwd: 5’-GGGATTCCCTGGACCTAAAG-3’ Rev: 5’-GGAACACCTCGCTCTCCAG-3’) and *GAPDH* (Fwd: 5’-AGCCACATCGCTCAGACAC-3’; Rev: 5’-GCCCAATACGACCAAATCC-3’) mixed with Power SYBR Green (Life Technologies) using the 7500 Fast Real-Time PCR System. Each sample was analyzed in triplicate, and the resulting data were averaged.

### Second Harmonic Generation (SHG) image acquisition and analysis

Imaging of fibrillar collagen in FDMs, obtained from fibroblastic/ECM units, was performed on a Leica SP8 confocal/multiphoton microscope (Leica Microsystems, Inc., Mannheim, Germany) as previously described^51^ by tuning the Coherent Chameleon Ultra II Ti:Sapphire laser (Coherent Inc., Santa Clara, CA) to 910 nm and collecting SHG signal on a nondescanned detector configured to capture wavelengths at 455 nm (20x (1.0 N.A) water immersion objective). Five images were taken for each MatTek dish (5 dishes per condition). Each image taken was 1024x1024 pixels (553.57 μm x 553.57 μm) with 20 z-stacks, 1 μm apart. Imaging parameters (laser power 80%, Gain: 85.1%, Offset: 75.4%) were kept identical between imaging sessions to allow for comparable image analysis quantification. The autofluorescence was subtracted from the original SHG images as previously described^52^. Imaging of fibrillar collagen in human biopsies was performed as above, but only one z-slice was taken per image.

### Collagen Analysis

Quantification of fibrillar collagen density from SHG images was calculated using ImageJ software as previously described^51^. Alignment was calculated using fast Fourier transform (FFT) as in^37^ and fiber orientation angles were analyzed with OrientationJ (ImageJ). The CT-FIRE program was used to quantify individual collagen fiber parameters (length, width and straightness) in every SHG image (http://loci.wisc.edu/software/ctfire, version 1.3, beta 2,^51^). For straightness, fibers were considered straight if the distance between fiber endpoints divided by the distance along the fiber is greater than the threshold value 0.92593 (1/1.08 = 0.92593) and the data are presented as the percentage of all fibers that were straight^52,53^.

### Proliferation, Apoptosis, and cell morphology of cancer cells within FDMs

Breast cancer cell lines MCF-7, MDA-MB-453 or MDA-MB-231 cells (2.4 × 10^4^/cm^2^) were plated on top of FDMs, prepared as described above, and cultured for 48 hours. For the apoptosis assay, media was changed after 48 hours and cultured for an additional 24 hours with media containing 1 μM Staurosporine (A8192, ApexBio Technology, Houston, TX). Cells were fixed with 4% paraformaldehyde (Electron Microscopy Sciences, PA) before proceeding to immunofluorescence staining (see below).

### Immunocytochemistry

Fixed cells were permeabilized with 100% methanol for 10 min at −20°C. Cells were blocked with 5% Bovine Serum Albumin, fraction V (BP1605-100; Fisher Scientific, NJ) and 0.3% Triton X-100 in PBS then incubated with primary antibodies: Ki67 (1:450, ab15580; Abcam), Active Caspase-3 (1:500, ab2302; Abcam) overnight at 4°C. Cells were washed before incubation with secondary antibodies (Alexa Fluor; Invitrogen, Grand Island, NY) and then washed and mounted with mounting medium containing DAPI (Vector Laboratories Inc.). Fluorescence was viewed using the Olympus BX51 microscope (Olympus, Melville, NY) as previously described ^37^. The proliferative or apoptotic index was calculated using ImageJ software (percentage of Ki-67 or Cleaved-Caspase-3 positive nuclei/total nuclei). For cell morphology, cells were stained with Alexa Fluor 555 Phalloidin (A12380, Invitrogen, ThermoFisher Scientific, CA) and DRAQ5 (Biostatus, United Kingdom), mounted with Fluoromount-G (SouthernBiotech, AL), and images were taken using a confocal microscope (see SHG imaging above).

### Cell Morphology Analysis

Fluorescent images stained with DRAQ5 (nucleus) and phalloidin (f-actin) were collected from four sets of experiments on siCtrl and siCol3 FDMs seeded with MCF-7 cancer cells. One representative experimental set is graphed (N=6 dishes per treatment). Automated image analysis was utilized to calculate morphometric parameters for individual cells from these images using the CellProfiler software (Ver. 3.1.9). Individual nuclei were identified from the DRAQ5 channel using the adaptive Otsu two class thresholding method with exclusion of objects based on sizes below 15 pixels in diameter (4 μm). Using the identified nuclei, cell bodies were identified using the phalloidin channel using propagation with minimum cross entropy thresholding. Primary and secondary objects were evaluated for morphometric parameters.

### 3D culture

3D cultures of human breast cancer cells (MCF-7) were established, as previously described^54^. MCF-7 cells, initially grown in 2D culture, were trypsinized and plated for 3D culture at 0.5 x 10^6^ cells per ml of growth factor depleted Matrigel (BD Biosciences, MA) supplemented with 200 μg/cm^2^ of human recombinant Col1 (C7624, Sigma-Aldrich), Col3 (ab73160, Abcam) or mixed Col1+Col3 (50:50) in 8-well Chamber slides. 200 μl of complete medium was added and assays were cultured at 37° for 10 days. Medium were subsequently changed every 2 days. Cells were stained as described above with E-cadherin primary antibody (610181; BD Biosciences, San Jose, CA) and DRAQ5. Images (>4 per well, >10 z-stacks (1.5μm thick)) were taken as described for FDMs. Spheroids, single cells, and nuclei area were identified from max z-projections of images using ImageJ software. Lumens were counted by examining each z-stack (E-cadherin and DRAQ5 staining).

### Tissue Immunofluorescence

Sections from fixed, paraffin-embedded human tissues were mounted onto charged glass slides. After deparaffinization and rehydration, antigen retrieval was performed by an incubation with proteinase K (20 μg/mL in Tris-ethylenediaminetetraacetic acid buffer). Sections were blocked in PBS (Odyssey Blocking Buffer; LI-COR, Lincoln, NE) containing 5% normal donkey serum. Sections were incubated with antibodies directed against Col3 (1330-01; Southern Biotech, Birmingham, AL), Col1 (PA5-9513; ThermoFisher Scientific), and Cytokeratin (M351501-2; Agilent, Santa Clara, Ca), followed by Alexa Fluor secondary antibodies (ThermoFisher Scientific) and DRAQ5. The slides were washed and mounted in fluorescent mounting medium (Fluoromount-G; SouthernBiotech). For collagen analysis, 20x images (one z-slice) were taken using confocal 2-photon microscopy^37^,^51^, see above. Three-five images per invasive and noninvasive regions were obtained from non-overlapping areas of interest containing both tumor cells and stromal collagen. These areas were identified on serial hematoxylin and eosin (H&E) stained histologic sections by a board-certified pathologist (EAM). Specifically, these regions were within the tumor mass, as opposed to the tumor periphery, capturing the multiple boundaries that occur throughout the tumor between islands of mammary carcinoma cells and local stroma. SHG images were simultaneously obtained.

For low magnification images to show gross morphology, slides were scanned using an Aperio Versa200 slide scanner (Leica Biosystems, Buffalo Grove, Illinois) and imaged for Col3, Col1, Cytokeratin, and DAPI (Penn Vet Comparative Pathology Core).

For mouse tissues, staining was performed as in human tissues, except citrate buffer antigen retrieval (HK086; BioGenex, Fremont, CA) was used, and DAPI was utilized to stain nuclei. Five non-overlapping images excluding necrosis were taken per tumor using an epifluorescent scope, as previously described^37^. Due to common cross-reactivity of collagen antibodies with other collagen types, the Col3 antibody (1330-01; Southern Biotech, Birmingham, Al) utilized was determined to be specific for Col3 based on immunoreactivity in young adult mouse (Col3^+/+^ and Col3^−/−^, 4 weeks old) dermis sections following the same protocol as human biopsies (see above).

### Impact of Col3 reduction and supplementation with Col3-containing hydrogels on tumor growth and recurrence

Orthotopic injections of 4T1 cells (50,000/fat pad) were performed into the fourth mammary fat pad of anesthetized 6-8 week-old, female BALB/C mice, as previously described ^37,47^. For the resection experiment Col3+/+ and Col3+/− mice were used. Tumors were marginally resected at 14 days post tumor cell injection, wounds closed with tissue adhesive (3M, Vetbond), and recurrent tumors were measured 14 days later. To examine if Col3-hydrogels inhibit tumor growth and aggressiveness, 4T1 injections were performed, as above, using our shear-thinning and self-healing injectable hydrogel system^55^. For the synthesis of shear-thinning hyaluronic acid (HA) hydrogels, sodium HA (60 kDa) was purchased from Lifecore Biomedical LLC (Chaska, MN) and all other chemicals were obtained from Millipore Sigma. First, HA was dissolved in deionized water at 2 wt%, exchanged against Dowex-100 resin, and neutralized by tetrabutylammonium hydroxide (TBA) to form HA-TBA^55^. Adamantane-modified HA (Ad-HA) was prepared via esterification by coupling 1-adamantane acetic acid to HA-TBA. For synthesis of β-cyclodextrin–modified HA (CD-HA), βCD-hexane diamine (CD-HAD) was formed by reacting β-cyclodextrin (CD) with a hexanediamine linker. CD-HDA was then prepared via amidation to HA-TBA. Percent modification of the HA backbone by adamantane (Ad-HA, 29%) and β-CD (CD-HA, 25%) was confirmed by nuclear magnetic resonance. Hydrogels of 3.5% total polymer concentration (wt/vol) were prepared by mixing stock solutions of Ad-HA and CD-HA in a 1:1 (CD-Ad) stoichiometric ratio in phosphate buffered saline.

In addition to cells, hydrogels were supplemented with 100μg of human recombinant Col3 (ab73160, Abcam, Cambridge, United Kingdom) or 5mM Acetic acid (A6283, Sigma-Aldrich, St Louis, MO) as vehicle (Ctrl). During the 4 weeks of the experiment, mouse weight and tumor sizes were measured 3 times per week. Tumor volume was calculated using the following formula: V = (L × W^2^)/2 as previously described ^37,56^. Mice were euthanized 28 days after tumor cell injection, and primary tumors and lungs were collected for additional analysis as previously described^37^.

### Mouse lung metastasis

Lungs from mice bearing tumors were perfused and fixed while maintaining inflation as described in^37^. To capture all metastases throughout the entire lung, the lungs were processed, inflated, embedded in paraffin and sectioned every 2mm. The 2mm sections were then re-embedded in paraffin blocks so that each block contained a cross section from each 2mm section of lung^57^. Blocks were sectioned, stained for H&E, and scanned in at 20x magnification (Penn Vet Comparative Pathology Core). ImageJ was used to identify metastases, which were confirmed by a board-certified pathologist (EAM), prior to quantification.

### TCGA breast cancer patient cohort data collection

For *in silico* analysis of Col1 and Col3 expression patterns, we used data generated from the breast invasive carcinoma (BRCA) cohort of The Cancer Genome Atlas (TCGA)^58,59^. Patient clinical annotations, bulk tumor sample RNA-seq gene expression data, bulk tumor ABSOLUTE purity estimates^60^, (PAM50 intrinsic mRNA expression subtype classifications from the most recent TCGA BRCA publication^61^, and curated patient survival endpoints^62^ for the TCGA BRCA cohort were downloaded from the National Cancer Institute’s Genomic Data Commons (https://portal.gdc.cancer.gov/). RNA-seq expression data was log2(x+1) scaled for downstream analysis.

### TCGA tumor purity correlation analysis and Col1:Col3 ratio calculation

Pearson correlations and corresponding p-values were calculated between ABSOLUTE tumor purity estimates and the gene expression estimates for the 20,531 unique genes included in the RNA-seq dataset for the TCGA BRCA cohort. Q-values were calculated by adjusting the p-values for false discovery rate using the Benjamini-Hochberg procedure.

Expression values for COL1A1 and COL1A2 were normalized by calculating the ratio of their expression to COL3A1 expression from the RNA-seq dataset for the TCGA BRCA cohort. Pearson correlations were then calculated between ABSOLUTE tumor purity and each of the COL1A1:COL3A1 and COL1A2:COL3A1 expression ratios, again correcting for multiple hypothesis testing with the Benjamini-Hochberg procedure.

### TCGA patient grouping and survival analysis of Col1:Col3 high/low patients

TCGA BRCA patients in the uppermost quartiles of the COL1A1:COL3A1 and COL1A2:COL3A1 expression distributions were classified as Col1:Col3 high patients and patients in the bottommost quartiles were classified as Col3:Col1 high patients. Patients in the middle two quartiles of both ratio distributions were classified as “other.” This roughly split the cohort into thirds with 372 Col3:Col1 high patients, 370 Col1:Col3 high patients, and 355 other patients. For comparison purposes, BRCA patients were also classified as COL1A1 high/low, COL1A2 high/low, and COL3A1 high/low using the same quartile-based approach.

### Data analysis

Values are expressed as means ± SD unless otherwise stated. Unpaired or paired t-tests were utilized to compare 2 groups, and 1-way ANOVAs followed by Tukey’s multiple comparisons tests were used to compare 3 groups. Tumor growth curves were compared via 2-way ANOVAs followed by Sidak’s multiple comparisons tests. Correlations were tested using Pearson correlation coefficient test. Outliers were removed if indicated by GraphPad Prism ROUT outlier analysis with α = 0.1. P-values <0.05 were considered statistically significant. Study groups were analyzed utilizing GraphPad Prism 9 statistical software except as stated below.

Correlations with RNA gene expression were calculated as Pearson correlations and adjusted using the Benjamini-Hochberg procedure. Two-sided Fisher’s exact tests were used to compare proportions of samples of each PAM50 mRNA expression subtype within the Col1:Col3 high and Col3:Col1 high groups. Kaplan-Meier curves for the Col1:Col3 high, Col3:Col1 high, and other patient groupings were calculated with Python using the lifelines survival analysis library (https://doi.org/10.5281/zenodo.1252342). Logrank tests were used to calculate p-values for comparing the survival distributions of the Col1:Col3 high and Col3:Col1 high patient groups. The survival curves and logrank tests were then repeated for the BRCA cohort using each of the COL1A1 high/low, COL1A2 high/low, and COL3A1 high/low classification schemes.

Kaplan-Meier curves for the Col1:Col3 high, Col3:Col1 high, and other patient groupings were calculated with Python using the lifelines survival analysis library (https://doi.org/10.5281/zenodo.1252342). Logrank tests were used to calculate p-values for comparing the survival distributions of the Col1:Col3 high and Col3:Col1 high patient groups.

## RESULTS

### Human Col3-deficient fibroblast/ECM functional units generate FDMs with enhanced tumor-permissive characteristics.

**Figure 1:**
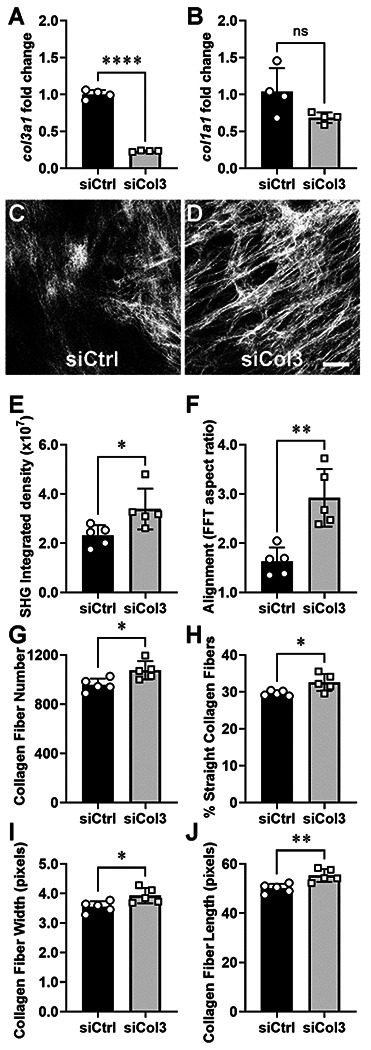
Col3 deficient-fibroblasts produce dense, highly aligned collagen matrices. Human skin fibroblasts were transfected with non-targeting siRNA (siCtrl) or Col3 siRNA (siCol3). After 96h, the efficiency and specificity of knockdown were confirmed by qPCR for col3a1 and col1a1 (A and B). Representative Second Harmonic Generation (SHG) images of collagen matrices from human fibroblast-derived matrices (FDM) prepared from fibroblast/ECM units treated with control and Col3-targeted siRNA (C and D). Collagen matrices were analyzed for total collagen amount (E) and collagen alignment (F). CT-FIRE was used to quantify collagen fiber number (G) straightness (H), width (I), and length (J) from SHG images. Data from representative experiments are presented (5 individual experiments performed with similar results). Error bars show mean ± SD (n = 5). Student unpaired t-tests: (*,p< 0.05; **,p < 0.01; ****, p<0.0001). Scale bar = 25 μm.

Given that a tumor-permissive stroma has been characterized by an increase in collagen density, alignment (e.g., anisotropy), and fiber characteristics including width, length and straightness^37,51,63,64^, we sought to determine if Col3 deficiency in human fibroblast/ECM functional units promoted deposition and remodeling of tumor-permissive fibrillar collagen matrices similar to those deposited by murine Col3^−/−^ fibroblasts^37^. To do so, we depleted COL3A1 in human fibroblasts using small interfering RNA (siRNA) against Col3 (*siCOL3A1*). Treatment of human fibroblasts with siCol3 for 96 h effectively reduced the mRNA levels of Col3 (~77% reduction) compared to siCtrl treated fibroblastic units, while there was no effect on Col1 mRNA expression (Figure 1A–B).

To determine the impact of Col3 deficiency in human fibroblasts on matrix deposition and organization, we used SHG microscopy to view collagen microarchitecture of the fibroblast-derived matrices produced by the siCol3 and the siCtrl fibroblasts. Collagen fibers appear less prevalent and organized in matrices deposited by fibroblasts treated with siCtrl compared to an increased collagen signal and fiber organization in matrices produced by those treated with siCol3 (Figure 1C–D). Subsequent image analysis confirmed an increased integrated density (Figure 1E) and fiber alignment (Figure 1F) in matrices produced by Col3-deficient fibroblast/ECM units. To analyze the characteristics of collagen fibers separately, we applied CT-FIRE and found that Col3-deficent fibroblasts produced fibers that were straighter, wider and longer than control fibroblastic units (Figure 1G–J). Together, these data are consistent with our previous *in vitro* and *in vivo* data supporting a role for Col3 in fibrillar collagen deposition and organization^37^, with conservation of this response in both human and murine fibroblast/ECM units.

### Col3-deficient matrix increases proliferation and decreases apoptosis of breast cancer cells.

**Figure 2:**
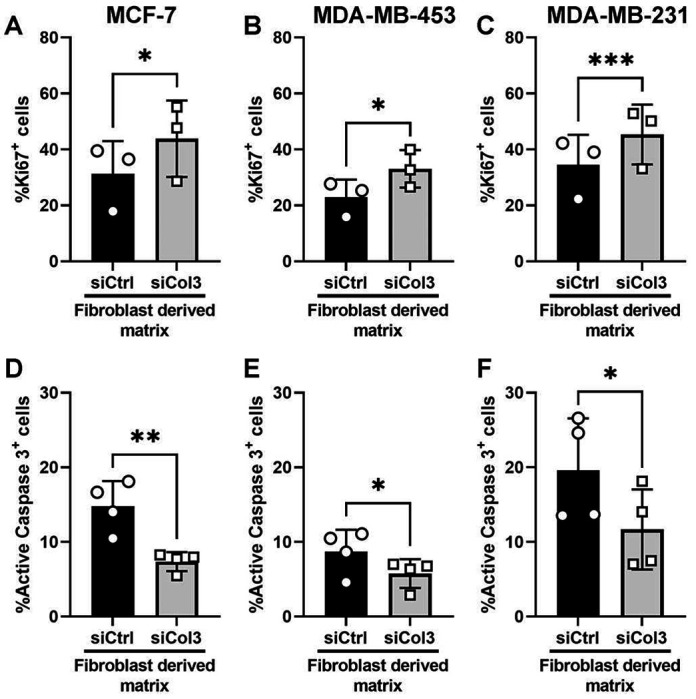
Col3-deficient matrices promote proliferation and inhibit apoptosis of breast cancer cells. Three breast cancer cell lines, MCF-7, MDA-MB-453 and MDA-MB-231 were cultured on collagen matrices produced by control fibroblasts (siCtrl) or Col3-deficient fibroblasts (siCol3). Cells were fixed and stained with Ki67, a cell proliferation marker or active caspase 3, a cell apoptosis marker. Quantification of the percentages of Ki67 (A, B, C), and active caspase 3 positive cells (D, E, F). Error bars show mean±SD (n = 3 for ki67, n = 4 for active caspase 3). Paired Student t-tests: (*,p<0.05; **, p<0.01; ***,p<0.001).

The interaction between cells and their surrounding microenvironment play an important role in regulating cell behaviors. To explore the effect of the tumor-restrictive and -permissive FDMs produced by siCol3 and siCtrl-treated fibroblast/ECM units, respectively, on human breast cancer cells, we assessed proliferation and apoptosis in three different cell lines that exhibit varying degrees of aggressiveness, ranging from a less-aggressive epithelial breast cancer cell line (MCF-7), to the moderately aggressive (MDA-MB-453) and highly-aggressive (MDA-MB-231) triple negative breast cancer cell lines. Cell proliferation and apoptosis were evaluated by immunostaining for Ki67 (a proliferation marker) and active caspase 3 (an apoptosis marker), respectively in cells grown on FDMs for 48 hours. Our results show a significant increase in percentage of Ki67 positive nuclei when cultured on FDMs produced by Col3-deficient fibroblasts, compared to control, independent of cancer cell line type (Figure 2A–C). Moreover, we identified a significant decrease (nearly 50%) in percentage of cells expressing active caspase 3 cultured on the FDMs produced by Col3-deficient fibroblasts in all three breast cancer cell lines, compared to control FDMs (Figure 2C–E). These findings are in accordance with our previous *in vitro* data using murine breast cancer cell line (4T1) on matrices produced by Col3^−/−^ embryonic fibroblast/ECM units and *in vivo* data using 4T1 tumors grown in Col3^+/−^ mice compared to Col3^+/+^ littermates; both experiments showing that reduced Col3 increased aggressive breast cancer cell phenotype^37^.

### Col3-deficient matrix affects MCF-7 cell and nuclear shape.

**Figure 3:**
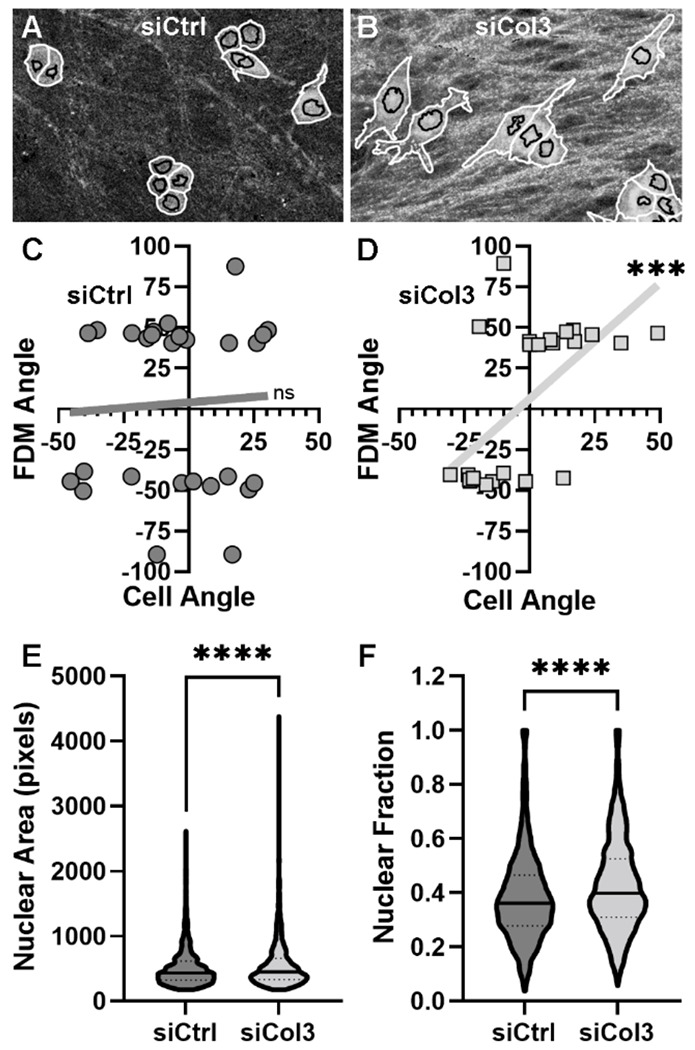
Col3-deficient matrices affect epithelial breast cancer cell morphology. Images of MCF-7 cells grown on FDMs and stained for phalloidin and DRAQ5 collected concurrently with SHG images. Cell segmentation was performed using CellProfiler software, using phalloidin as a cell shape marker and DRAQ5DRAQ5 as a nuclear marker. CellProfiler cell identification is shown as white cytoplasmic and black nuclear outlines over SHG signal (grey) (A-B). Scale bar = 25 μm. Collagen fiber angles (mode of collagen fiber angle histogram, OrientationJ) from the FDMs were analyzed and compared to cell orientation angles. Lines shown are linear regression. Pearson correlation: ***p<0.001, C-D). Quantification of nuclear area and nuclear fraction (area of nuclei/total cell area). Solid lines at median and dotted lines at SD (n > 1500 analyzed cells per treatment, student t-test with Welsh correction: ****p<0.0001, C-D)).

Given the well-known effect of ECM topography on cell behaviors^65^, we explored the impact of loss of Col3 in FDMs on morphology of MCF-7 cells. To characterize the impact of the Col3-deficient matrix on cancer cell morphological changes, cellular and nuclear features, including cell and nuclear area, cell compactness (a measure of cell irregularity), cell orientation and nuclear fraction, were quantitated using CellProfiler software analysis of phalloidin and DRAQ5 stained images^66^, identifying the cytoskeleton and nuclei respectively (Fig 3A–B). When the angles of the collagen fibers in the matrix were compared to the MCF-7 cells’ orientation angles, we found that the cells grown on siCol3 matrix orientated themselves with the collagen fibers, while the cells on siCtrl matrix did not (Figure 3C–D). Additionally, we found that the nuclear area and nuclear fraction, key indicators of tumor cell aggressiveness^67^ were increased in cells grown on siCol3 matrix (Figure 3E–F). The data also show that while the cells grown on siCtrl matrix were larger in area (p=0.007), they were also more compact (p<0.0001) than those grown on siCol3 matrix (data not shown), suggesting a less aggressive phenotype^68^. These modifications potentially reflect greater cell epithelial to mesenchymal transition (EMT) behavior of cells in a Col3-depleted environment^67,69^.

### Decreased Col3:Col1 ratios correspond with tumor-permissive stroma and invasive tumor regions in human TNBC biopsies.

**Figure 4:**
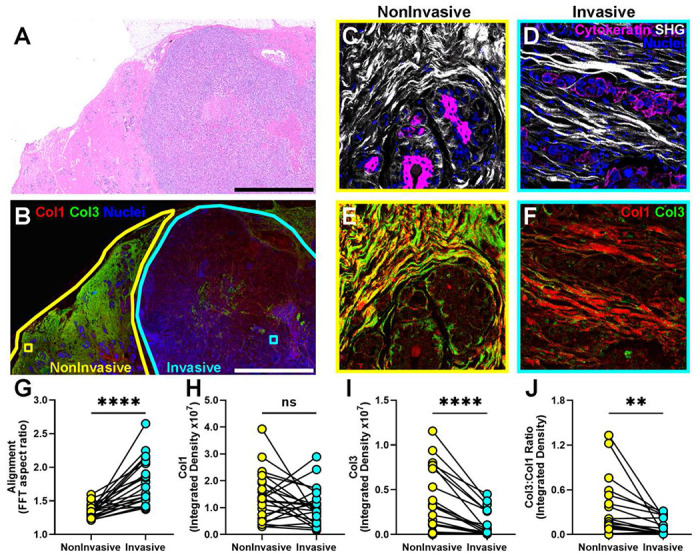
Decreased Col3:Col1 ratio as a potential biomarker of tumor-permissive stroma. Hematoxylin-Eosin images were used to identify invasive and noninvasive regions within the same human triple-negative breast cancer (TNBC) biopsy. (A-B) A representative image of a human TNBC biopsy was immunolabeled for Col1 (Red), Col3 (Green), and stained with the nuclear stain DRAQ5 (Blue) (B). Second harmonic generation microscopy (SHG, white; Cytokeratin, magenta; DRAQ5/nuclei, blue; C-D) images were combined with confocal fluorescent imaging (Col1, red; Col3, green; E-F). SHG images were used to measure fiber alignment (FFT aspect ratio, G). The positive immunoreactivity of Col1 and Col3 integrated density of in both invasive and noninvasive regions, and their ratio, was analyzed using ImageJ (H-J). N=23 tumors. Paired Student t-tests: (**p<0.01, ****p<0.0001). Scale bar = 4mm.

Given our data and recent data from other groups suggesting a tumor-suppressive role for Col3 in breast cancer, we next sought to resolve conflicting data in the literature regarding the use of Col3 as a prognostic marker in breast cancer biopsies. To assess whether tumor-permissive and -restrictive architectural features of fibrillar collagen and specific localization of Col1 and Col3 associate with invasive and noninvasive regions of breast cancer patient biopsies, we used a combination of SHG and immunofluorescence imaging of pathologist-defined invasive and noninvasive regions within hematoxylin and eosin-stained sections of each biopsy in 22 human triple-negative breast cancer samples (Figure 4A). We used SHG microscopy to visualize fibrillar collagen combined with immunofluorescence to visualize nuclei (DRAQ5; Figure 4B–D) and cytokeratin-positive epithelial (tumor; Figure 4C–D) cells, as well as distribution of Col1 and Col3 (Figure 4B, E, F and Supp. Figure S1). Consistent with previous reports supporting the association between aggressive cancer behavior and collagen alignment^51,52,63,70^, we found that invasive regions had significantly more aligned fibers compared to noninvasive regions (Figure 4G). As expected, the amount of Col3 negatively correlated with collagen fiber alignment (Supp. Figure S2). The fibrillar collagen SHG signal showed that noninvasive regions generally had short, wavy and non-aligned fibers surrounding islands of tumor cells (Figure 4C, D). Although analysis of 3-5 non-overlapping images within these invasive and noninvasive regions revealed no significant difference in Col1 staining between regions, noninvasive (less aggressive) regions of tumors were characterized by significantly greater immunoreactivity for Col3 compared to invasive regions (Figure 4H and I; p<0.001). Furthermore, we found that the Col3:Col1 ratio was significantly higher in noninvasive regions (Figure 4J), suggesting that Col3 was associated with tumor-restrictive stroma. Based on these data, and in accord with our above-presented *in vitro* data, we suggest that lower absolute and relative interstitial matrix levels of Col3 are a marker for tumor-permissive stroma and more aggressive tumor behavior.

### In silico screening identifies breast cancer patients with higher ratio Col3:Col1 bulk tumor expression have improved disease-free and progression-free survival.

Based on these results, we hypothesized that an *in silico* approach designed to examine relative Col3 expression levels in bulk tumor samples would confirm that higher relative Col3 expression associates with a less aggressive clinical course. Given the evidence that different Col1:Col3 ratios correspond to different tissue perturbation states^71,72^ and should similarly vary during a desmoplastic stromal response, we hypothesized that using a Col1:Col3 gene expression ratio instead of Col3 expression alone would (i) isolate a more biologically relevant gene expression metric for analysis that accounts for rise in fibrillar collagens during the desmoplastic response and (ii) address the heterogeneity of bulk tumor biopsies by normalizing a stromal signal to a co-expressed stromal signal for each sample, thereby correcting for varying stromal content between samples – a potential source of bias that prior analyses of collagen expression in bulk tumor data have not accounted for.

To validate this, we used generated data from 1,099 breast cancer patient tumors in the TCGA BRCA cohort. We demonstrated that the estimated fraction of cancer cells in each bulk tissue sample (i.e. tumor purity) significantly associated with the expression of Coll and Col3 transcripts with increased levels of tumor content corresponding to decreased expression of COL1A1, COL1A2, and COL3A1 (Supp. Fig. S3), highlighting how variable sample content may bias patterns observed during bioinformatic analysis of tumors. Conversely, when using COL1A1:COL3A1 and COL1A2:COL3A1 expression ratios to evaluate for associations with tumor purity, we found a substantially diminished relationship (Supp. Fig. S4). Unlike the individual collagen gene measures, these expression ratios appeared to be significantly demarcated from stromal fraction biases, as pathway analysis of gene expression correlates found significant enrichment of ECM-related pathways within the top individual COL1A1, COL1A2, and COL3A1 expression correlates, but not within the top COL1A1:COL3A1 and COL1A2:COL3A1 expression ratio corrrelates (see Supplementary Material).

**Figure 5:**
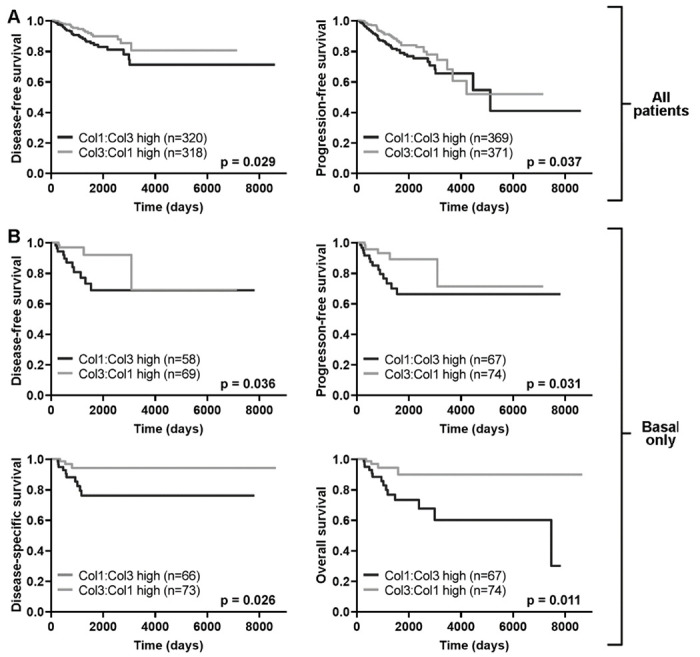
The Col3:Col1 expression ratio significantly associates with clinical outcome. A. Kaplan-Meier survival curves showing that Col3:Col1 high patients have significantly better prognosis than Col1:Col3 high patients in terms of DFS and PFS across the entire TCGA BRCA patient cohort (p<0.05). B. Patients with Col3:Col1 high basal tumors demonstrated significantly better DFS, PFS, DSS, and OS compared to those with Col1:Col3 high basal tumors (p<0.05).

To test whether higher or lower relative levels of tumor Col3 associated with clinical outcome, we then separated TCGA BRCA patients into those with higher Col3:Col1 ratio tumors and those with lower Col3:Col1 (i.e. higher Col1:Col3) ratio tumors (see Methods for classification criteria). Interestingly, two-sided Fisher’s exact tests revealed no association between PAM50 mRNA expression subtype and Col1:Col3 ratio classification, suggesting that breast tumors of any intrinsic subtype are equally likely to be Col1:Col3 high or Col3:Col1 high. When evaluating Kaplan-Meier survival curves for the Col1:Col3 high and Col3:Col1 high patient groups, we found that Col3:Col1 high patients demonstrated significantly better prognosis than Col1:Col3 high patients in terms of both disease-free and progression-free survival (p<0.05, logrank test) (Figure 5A). When the survival analysis was repeated while stratifying the cohort according to PAM50 subtype, patients with basal tumors (which encompass most of the triple-negative breast cancer cases) had uniform directionality when examining all four available clinical endpoints: those with Col3:Col1 high basal tumors had significantly improved disease-free, progression-free, disease-specific, and overall survival compared to those with Col1:Col3 high basal tumors (p<0.05, logrank test) (Figure 5B). A table summarizing the number of Col1:Col3 high and Col3:Col1 high patients with available data for each clinical endpoint and the accompanying logrank test p-values is shown in Supp. Fig. S5.

When this survival analysis was replicated using high-expressing and low-expressing patient groups classified according to COL1A1 expression alone, COL1A2 expression alone, and COL3A1 expression alone, the resulting survival curves for the COL1A1, COL1A2, and COL3A1 high-expressing patients resembled one another in shape and directionality, as did the low-expressing patient survival curves (Supp. Fig. S6, S7, S8). Though not reaching significant thresholds, the curves calculated across all patients suggested that higher expression of each collagen transcript trended towards worse disease-free and progression-free survival. This re-emphasizes how mining for associations between patient outcome and single stromal elements measured from bulk tumor samples can yield results that are subject to influence by inter-sample variation in stromal content and cancer cell fraction.

### 3D culture: Col3-enriched microenvironment limits MCF-7 migration and enhances lumen apparition.

**Figure 6:**
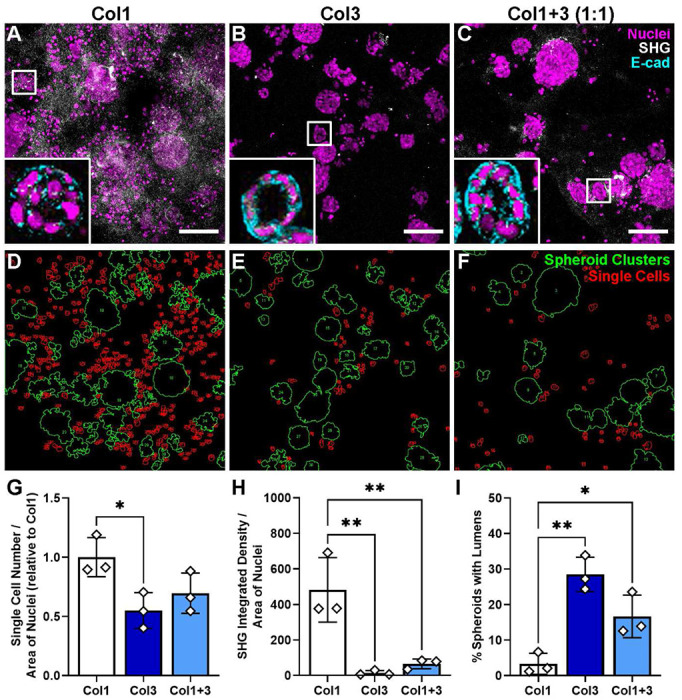
Increasing exogenous-Col3 in the 3D culture microenvironment promotes a benign phenotype in epithelial breast cancer cells. MCF-7 cells were grown in 3D-culture consisting of Matrigel supplemented with human recombinant Col1, Col3 or 50:50 Col1+Col3 for 10 days. Images were taken with confocal microscopy of nuclei (DRAQ5, magenta), E-cadherin (cyan, shown in inset only) and fibrillar collagen (SHG, white) on max projection images. Mid-sections of the images showed the apparition of lumens (insets) (A-C). Spheroids (green outlines) and single cells (red outlines) were identified using ImageJ (D-F). Quantification of the area of single cells normalized to the total nuclei area (G). Integrated density of collagen fibers was analyzed by ImageJ and normalized to the total nuclei area (H). Lumens were counted and normalized total spheroids (I). Error bars show mean ± SD (n = 3). Data from three independent experiments. One-way AVONAs followed by Tukey post-hoc tests: (*, p<0.05; **,p<0.01,). Scale bar= 100μm.

Given that higher Col3:Col1 ratios were associated with a tumor-restrictive stroma and longer survival in human patients, we next tested the hypothesis that adding exogenous Col3 would suppress aggressive breast cancer cell behavior utilizing a 3D collagen culture system ^54^ where we modulated the Col3:Col1 composition of the matrix by adding human recombinant Col1, Col3 or a 1:1 mixture of both. MCF-7 cells were grown in the 3D cultures for 10 days, then fixed and stained for DRAQ5 (to identify nuclei/cells) and E-cadherin, and imaged for SHG (Figure 6A–F). Overall, the majority of cells formed spheroids in all three conditions but in cultures containing Col1 without Col3, there were nearly twice the number of single cells between the spheroids compared to cultures containing Col3, with an intermediate number of single cells in cultures containing the mixture of Col1 and Col3 (Figure 6D–G; p<0.05). These data showed the involvement of the ECM composition on tumor cell morphology and suggest their impact on outward cell migration of the epithelial breast cancer cells from clusters of breast cancer cells.

Additionally, using SHG to image fibrillar collagen, there was >30-fold increase in SHG signal noted in Col1 only containing cultures compared to those containing Col3 (Figure 6H; p<0.01). Inclusion of Col3 with Col1 also suppresses SHG signal >7-fold compared to that in cultures containing Col1 alone (p<0.01). These findings support our previous data (^37^ and Figure 1) that Col3 inhibits the ability of the either stromal or cancer cells to remodel the matrix. These findings led us to hypothesize that the presence of Col3 in the microenvironment could limit the evolution of a tumor-permissive collagen matrix and suppress aggressive phenotype in breast cancer cells. In support, in the presence of Col3, we noted a greater number of spheroids containing a lumen in the mid-section of the spheroids (Figure 6A–C insets). The spheroids appeared as well-polarized epithelial monolayer surrounding single hollow lumens in Col3 containing cultures. Quantification of this effect showed an 8.6-fold induction of lumen formation (p<0.01) in presence of Col3 alone and 5-fold in the presence of equal amounts of Col1 and Col3 in the cultures (p<0.01). As normal breast epithelial cells form lumens in 3D culture, our data showing that Col3 can induce lumen formation in nearly one-third of the spheroids suggest that Col3 can promote a phenotype reminiscent of normal, non-cancerous epithelium. Our data show that Col3 promotes a tumor-suppressive microenvironment, limits breast cancer aggressiveness by promoting a more benign phenotype when increased in the tumor microenvironment.

### Col3-enriched hydrogels limit tumor growth and lung metastasis.

To determine how Col3 levels may regulate the evolution of a feed-forward tumor-permissive microenvironment and if increased Col3 in the tumor microenvironment limits local recurrence, we examined Col3:Col1 expression and post-resection local recurrence in wild-type (Col3^+/+^) and Col3 haploinsufficient (Col3^+/−^) mice either alone or in the presence of Col3-containing hydrogels. Specifically, tumor growth and metastasis were examined in a murine model of breast cancer in which tumor cells were orthotopically injected using a hydrogel as a carrier in the presence or absence of co-delivered exogenous Col3.

**Figure 7:**
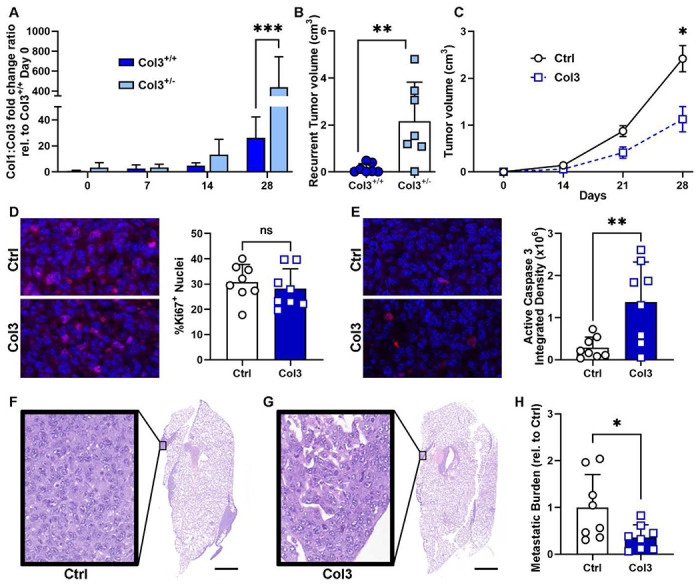
Use of Col3-enriched hydrogels promote a tumor-restrictive microenvironment in vivo. RNA was collected from 4T1 tumors in Col3^+/+^ and Col3^+/−^ mice and analyzed for Col1 and Col3 expression. Fold changes to Col3^+/+^ D0 samples were used to generate Col1:Col3 ratios (A). Tumor volume 14 days after marginal resection in Col3^+/+^ and Col3^+/−^ mice (B). Hydrogels were prepared with murine TNBC-like breast cancer cells (4T1) and supplemented with 100 μg of human recombinant Col3 or acetic acid (Ctrl) prior to injection into mammary fat pads of BalbC mice and harvested after 28 days (C). Tumor sections were stained for DAPI (nuclei, blue) and Ki67 (proliferating nuclei, red, D) or active caspase 3 (apoptotic cells, red, E). Scale bar = 50 μm. Staining was quantified as % Ki67 positive nuclei (D) or total active caspase 3 staining (integrated density, E). Lung sections were stained with H&E (F-G) and gross pulmonary metastasis was quantified (H). Scale bar = 3mm. For tumor growth, 2-way ANOVA followed by Sidak’s post-hoc test; for Ki67, active caspase 3, and metastasis, unpaired student t-tests: (*,p<0.05; **,p<0.01).

Given the striking difference in Col3 levels in noninvasive and invasive regions of human breast cancer biopsies (Figure 4), we hypothesized that a tumor microenvironment that is initially deficient in Col3 may evolve into a progressively more tumor-permissive microenvironment with a corresponding increase in Col1:Col3 ratio over time. To address this hypothesis, we examined Col1:Col3 mRNA expression in orthotopic 4T1 tumors in Col3^+/+^ and Col3^+/−^ mice over time. Given that primary tumors in Col3^+/−^ mice grow and invade the surrounding structures more readily and metastasize to a greater extent than in Col3^+/+^ littermates and that invasive regions in human TNBC biopsies contained a high Col1:Col3 ratio (Figure 4I), our data showing a dramatic increase in Col1:Col3 ratio as the tumor permissive stroma develops in Col3^+/−^ mice (15-fold seen in tumors of Col3^+/+^ mice at day 28 vs 2.8 fold at day 21) reveals how Col1:Col3 content in invasive and noninvasive regions may evolve temporally (Figure 7A).

Taken together that collagen alignment, which is negatively associated with Col3 levels but positively associated with recurrence in breast cancer patients, and that Col3 has been previously reported to maintain dormancy in cancer cells^48^, we next sought to determine whether this Col3-deficient microenvironment would drive local recurrence following marginal resection of orthotopically injected tumors. To do so, 4T1 orthotopic tumors were established for 14 days in Col3^+/−^ mice and their wild-type littermates and marginally resected. Following 14 days of post-resection healing, recurrent tumor burden was assessed and quantitated as previously described^37^. Not only was the incidence of local recurrence increased in Col3 deficient mice following marginal resection, but the volume of recurrence dramatically increased with a mean volume over 14-fold (Figure 7B; p<0.01) greater than in wild-type littermates post-resection.

As previously proposed and based on our data supporting a tumor-suppressive role for Col3 and its ability to direct a wound healing response post-resection^37^, we hypothesized that Col3-containing hydrogels could be used to engineer a tumor-suppressive microenvironment to treat residual microscopic disease. To test the hypothesis whether clinically applicable hydrogels could suppress tumor growth of microscopic disease and potentially limit distant metastasis, a fixed number of 4T1 cells in hydrogels supplemented with recombinant Col3 or vehicle control (Ctrl) were injected orthotopically in BALBc wild-type mice and tumor growth was monitored for 4 weeks. These studies used a class of hyaluronic acid (HA) hydrogels that form through noncovalent guest–host interactions, undergo disassembly (shear-thinning) when injected through a syringe and then reassemble within seconds (self-healing) when shear forces are removed^55^. These hydrogels allow for injection of breast cancer cells with the Col3 as part of their microenvironment during early tumor establishment; however, they could also be used to “coat” post-resection sites prior to surgical closure. By day 28, the tumors were significantly larger (~50% in volume) in Ctrl treated compared to Col3 hydrogels (Figure 7C). These findings are in accordance with our *in vitro* study confirming that a Col3-enriched microenvironment promotes a tumor-suppressive stroma for breast cancer cells.

Previously, we found that Col3 within the microenvironment affects tumor cell proliferation and apoptosis (Figure 2). To determine if the growth difference in tumors supplemented with Ctrl vs Col3 was due to proliferation and/or apoptosis, tumor sections were stained and imaged for Ki67 and active caspase 3. We saw no difference in proliferation (Figure 7D) but observed that Col3-supplemented hydrogel tumors had significantly more (over 4-fold) active caspase 3 staining (Figure 7E), suggesting that Col3 limited tumor growth through promoting tumor cell apoptosis.

Finally, we assessed if Col3-supplemented tumors metastasized to the lung to the same degree as control 4T1 tumors. Lung sections were stained for H&E (Figure 7F–G), evaluated for metastases by pathologists (EAM, DG), and metastatic burden was quantified using ImageJ. Mice with Col3-supplemented tumors had limited pulmonary metastasic burden, compared to those with control tumors (Figure 7H), further supporting our hypothesis that Col3 limits aggressive tumor cell behavior and supporting our hypothesis that Col3 hydrogels could be used to engineer a recurrence-suppressive post-resection tumor microenvironment^37^.

## DISCUSSION

The present study investigates the role of Col3 in breast cancer to deepen our understanding of the mechanisms by which it regulates cancer behaviors, how it may be utilized as a prognostic marker and provide proof-of-concept for its use as a therapeutic to limit recurrence. Our results show that Col3 prevents a switch to a tumor-permissive microenvironment through its effects on fibrillar collagen deposited and remodeled by stromal fibroblasts/ECM functional units and that loss of Col3 drives formation of tumor-permissive matrices that are capable of driving aggressive behaviors in multiple breast cancer cell lines. Notably, our work resolves a major discrepancy in the literature regarding the prognostic information associated with Col3 expression in biopsy samples by considering tumor heterogeneity, desmoplastic responses, and matrix architecture. Finally, our data provides proof-of-concept that Col3 hydrogels in post-resection surgical sites of breast cancer patients may improve clinical outcomes by suppressing recurrence.

The interactions between breast cancer cells and their tumor microenvironment plays a critical role in the tumor progression. Multiple studies show that dysregulation of the breast ECM such as increased stromal collagen deposition, as well as the organization and stiffness of the collagen matrix are correlated with increased breast cancer risk and poor prognosis and initiate mammary tumor growth and invasion ^14,37,51,52,63,64,73–79^. While our study focuses on Col3, it is important to note that Col3 has been established to play a crucial role in the fibril network formation of other fibrillar collagens ^32,33,80,81^. Our previous studies have revealed a role for Col3 in regulation of fibroblast/ECM functional unit activation and subsequent collagen deposition to promote scar formation and desmoplasia in part by regulating matrix microarchitecture ^34,37,47^. These different roles of Col3 and implications for the tumor microenvironment led us to explore more specifically Col3’s characteristics such as composition, organization, and distribution in the breast tumor microenvironment using multiple approaches (*in vitro, in vivo* and *in silico*). Consistent with our findings in murine Col3-deficient fibroblasts and our previously demonstrated tumor-restrictive role for Col3^37^, we show herein that human fibroblasts deficient in Col3 produce a denser, more aligned matrix comprised of straighter, wider and longer collagen fibers. These microarchitectural features have been associated with poor prognosis in both human and veterinary patients with mammary cancer^51,52,63,82^. The inverse relationship between collagen alignment, a robust predictor of aggressive behavior including recurrence, and Col3 levels is also established in human biopsy samples.

To date, the literature related to the role of Col3 in the breast cancer microenvironment remains conflicted with both reports on its associations with both tumor-permissive and tumor-restrictive behaviors^28,37,38,47,48,83–85^. With respect to the association of Col3 with malignant behavior and prognosis in breast cancer patients, we hypothesized that regional differences in Col3 and Col1 composition within a single tumor might obscure associations between collagen type and biologic influence or that an absolute rise in Col3 associated with a desmoplastic response in breast cancers attributed to a tumor-permissive role. To address this limitation, we exploited the heterogeneity within tumors to examine Col3 and Col1 content within pathologist-defined invasive and noninvasive regions of TNBC biopsies. Accounting for phenotypic heterogeneity, we show that absolute Col3 as well as relative Col3 (Col3:Col1) immunoreactivity was significantly associated with noninvasive regions of these biopsies. In fact, the degree of enrichment or depletion of Col3 relative to Col1 regionally in tumor-restrictive and - permissive areas within biopsies, respectively, was striking. Using the murine orthotopic 4T1 model, our data revealed how Col3-deficient tumors evolve over time with exponentially increasing Col1:Col3 expression levels, whereas Col3-sufficient mice, bearing less aggressive tumors, have a much more attenuated response. These results suggest a feed-forward regulation in response to loss of Col3 and its secondary tumor-permissive microenvironment in breast cancer.

In prior studies that examined associations between fibrillar collagen expression and human breast cancer behavior *in vivo*, Col3 expression has been associated with both more and less aggressive tumor behavior. Several papers have shown that an increase in Col3 expression at both the transcriptional and translational level are associated with malignancy and patient metastasis^28,83,84^, while others have suggested a tumor-suppressive role for Col3 in human breast cancer biopsies and *in vivo* models. Similar to the lack of intratumor homogeneity, there also exists a lack of intertumor homogeneity that previous studies have failed to account for when evaluating associations with Col3 expression. Given that the mutational landscape and cellular composition vary within tumors^45,86,87^ and sampling methods commonly used for processing tumor biopsies ignore spatial biases within tumors^88^, each tumor sample represents an admixture of cancer cells and non-cancer cells, including immune/inflammatory cells, endothelial cells, CAFs, and others in unknown fractions ^45^. However, given the fact that Col1 and Col3 have been reported to increase during breast cancer progression as part of the reactive stromal response^89,90^, increased Col3 levels may be coincident with the transition to malignancy. In recognition of this, we demonstrate that gene expression levels of the Col1 and Col3 monomer transcripts significantly correlate with the estimated proportion of non-cancer cells within samples of the TCGA BRCA cohort, suggesting that non-uniformity between breast tumors are a strong source of bias during *in silico* evaluation of Col3 gene expression. However, we also show that this bias can be alleviated by normalizing Col3 gene expression by Col1 gene expression, providing a metric for unbiased evaluation of relative Col3 levels in tumors. The approach of using a Col3:Col1 expression ratio when considering bulk tumor samples is favorable for two reasons. First, it normalizes Col3 gene expression using genes that are largely co-expressed by the same cell types that are found in the stroma: mesenchymal cells such as fibroblasts, myofibroblasts, and their progenitors ^91–94^. Second, it transforms Col3 expression into a biologically relevant signal that may provide a new context for understanding its function.

Using this approach to determine if relative Col3 could provide prognostic information regarding breast cancer patient survival, we found that patients classified as Col3:Col1 high fared better in terms of DFS and PFS than patients classified as Col1:Col3 high. These data are consistent with our observation that malignant tumor cells exhibit more aggressive growth in environments where Col1 is preferentially enriched relative to Col3 *in vitro* and our data showing increased Col3 expression in noninvasive regions of women’s breast cancer biopsies. Therefore, we propose that for bulk tumor expression levels, the ratio of Col1:Col3 may provide superior prognostic information. A large body of work now supports that the influence of fibrillar collagens on cancer and stromal cell behavior in the breast cancer microenvironment is not directed by quantitative differences alone but is also influenced by type, organization, and post-translational modification as well. Our work^37,47^ and that of others ^48^ has revealed direct tumor-restrictive effects of Col3 on cancer cell behaviors, which appear to be influenced by Col3 and its profound influence on fibrillar collagen organization. Given the association with collagen alignment and recurrence in breast cancer patients^63,82^, our data showing that Col3:Col1 ratio is inversely correlated to collagen alignment is consistent with its tumor-suppressive role.

We have previously shown that Col3 is a critical component of the healing niche of cutaneous wounds and that its loss increases scar formation by increasing myofibroblast persistence^34^. Dvorak first referred to tumors as wounds that do not heal, noting the similarities between healing and tumor microenvironments ^95,96^. Consistent with this concept, we found that Col3^+/−^ mice develop significantly more local recurrence than Col3^+/+^ littermates. Conversely, we hypothesized that we could exploit the vulnerary effects of Col3 on healing and its tumor-restrictive properties in the post-resection healing environment to decrease aggressive cancer behaviors in the 4T1 orthotopic model in Col3-sufficient (wild-type) individuals. The ability of Col3 supplementation to decrease aggressive phenotype in our 3D cultures provided support for this hypothesis. Notably, we showed that both tumor growth and lung metastatic burden are decreased in individuals in which Col3 was increased in the early TME. Although we previously found that orthotopic (4T1) tumors in Col3-haploinsufficient (Col3^+/−^) mice exhibited increased proliferation compared to those in wild-type littermates, we did not observe a decrease in proliferation in tumors formed in a Col3 enriched environment. Consistent with our previous work showing Col3 loss decreased apoptosis within tumors, we did see a significant increase in apoptosis in tumors developing in the presence of Col3-supplemented hydrogels. Given the Col3-induced changes in matrix microarchitecture, Col3 may lower resistance to apoptosis through its effects on cancer cell mechanotransduction. These effects may provide synergy with other therapies, such as chemotherapy, to improve control of residual microscopic disease. Hydrogels for oncologic reconstruction currently serve two purposes: to improve healing and to deliver anti-cancer therapies. While numerous reconstructive hydrogels have been on the market to improve healing and aesthetic outcomes following mastectomy and breast conserving surgery, polymer-based chemotherapy delivery systems to reduce local cancer burden have also been more recently approved by the FDA^97–103^. Unfortunately, such chemotherapy delivery systems can negatively impact healthy cells in surrounding tissue, impeding the healing process. Although preliminary in nature, our data suggest Col3 hydrogels have the potential to suppress recurrence, reduce distant metastatic burden and improve healing of surgical sites, and thus may be uniquely advantageous for therapeutic application in breast cancer patients.

Taken together, these data collectively suggest that Col3 indeed has a tumor-suppressive role in breast cancer and provide proof-of-concept that Col3-based tissue engineering strategies may prove a safe and effective modality for limiting recurrence and improving care of breast cancer patients.

## Supplementary Material

1

## Figures and Tables

**Table 1.** Stage and Grade of Human Ductal Carcinoma Biopsy Samples (N=23 Total)

=N per Stage/Grade	*Grade*	
Stage	II	III
**I**	0	9
**II**	1	10
**III**	0	3

## Data Availability

The datasets generated during and/or analyzed during the current study are available from the corresponding author on reasonable request.

## Abbreviations

BRCA: Breast Cancer
CAF: Cancer-associated fibroblasts
Col1: Type I collagen
Col3: Type III collagen
ECM: Extracellular Matrix
EMT: Epithelial mesenchymal transition
FDM: Fibroblast derived matrix
FFT: Fast Fourier Transform
HA: Hyaluronic acid
SHG: Second Harmonic Generation
siRNA: Small interfering RNA
TCGA: The Cancer Genome Atlas
TME: Tumor microenvironment
TNBC: Triple-negative breast cancers
