# Supplementary material for: Tumor-restrictive type III collagen in the breast cancer microenvironment: prognostic and therapeutic implications
Source: Res Sq. 2023 Apr 12:rs.3.rs-2631314. Preprint. [Version 1] doi: 10.21203/rs.3.rs-2631314/v1 (PMC10120781; doi:10.21203/rs.3.rs-2631314/v1)
Supplement: 1 [file NIHPPrs2631314v1-supplement-1.pdf]

Supplementary Data

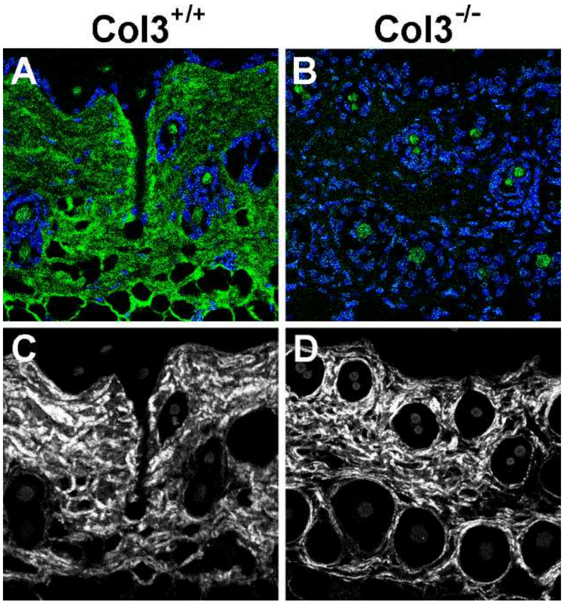

Figure S1: Specificity of Col3 antibody in Col3$^{-/-}$ mouse dermis. *Col3$^{+/+}$ and Col3$^{-/-}$ Mouse skin was immunostained for Col3 (green), stained with DAPI (nuclei, blue; A-B) and SHG (white) imaging was simultaneously obtained (C-D). Col3$^{-/-}$ dermis lacks immunostaining (hair shafts exhibit autofluorescence).*

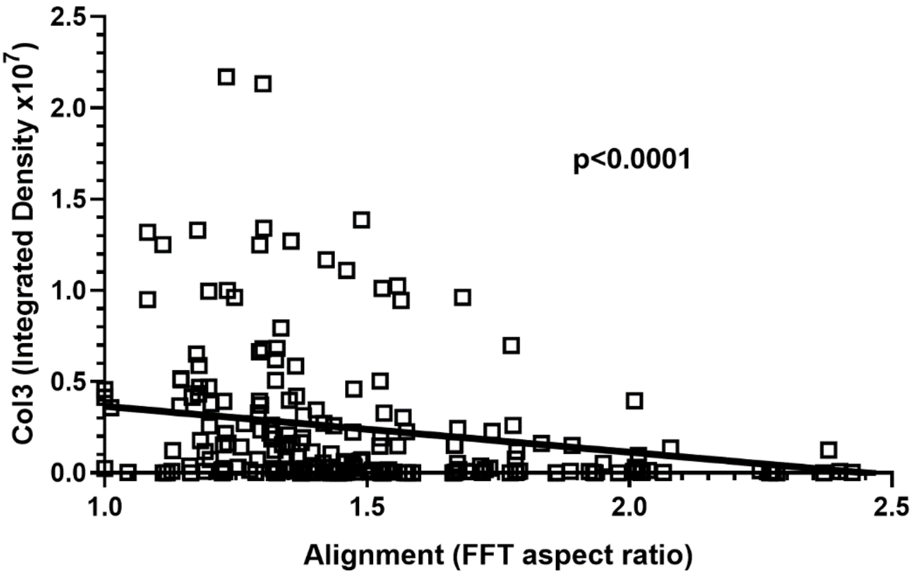

Figure S2: High Col3 staining is associated with non-aligned collagen fibers. *Human biopsy images were stained for Col3 and SHG images were simultaneously obtained. Col3 staining (integrated density) was calculated and plotted with collagen fiber alignment (SHG FFT aspect ratio) for each image obtained (N=23 tumors; 7-10 images per tumor). Linear regression and Pearson coefficient was used to test for correlation.*

Resolving issues of tumor biopsy heterogeneity in silico analysis of Col1 and Col3 expression in human breast cancer

When using our bioinformatics approach to mine for associations between Col3 expression and patient clinical outcome, we aimed to address the issue of varying stromal content between patient tumors – a potential source of bias when analyzing RNAseq-derived gene expression measurements of the Col1 and Col3 monomers in the TCGA BRCA bulk tumor samples. To identify this bias, we calculated correlations between estimated primary tumor purity values (representing the estimated proportion of cancer cells within samples) and gene expression of COL1A1, COL1A2, and COL3A1 and found that each gene demonstrated significant inverse correlations with tumor purity estimates across the entire BRCA cohort ($p<0.0001$, Pearson correlation coefficient t-test) (Supp. Fig. S3A). To compare tumor purity values across the intrinsic mRNA expression-based breast cancer subtypes, we stratified the BRCA cohort by PAM50 subtype classification and found that tumor purity values significantly varied between subtypes ($p<0.0001$, Kruskal-Wallis H test) (Supp. Fig. S3B). Furthermore, when COL1A1, COL1A2, and COL3A1 gene expression were stratified by PAM50 subtype, they were found to significantly vary ($p<0.0001$, Kruskal-Wallis H test) in a pattern inverse to that of tumor purity (Supp. Fig. S3C). The collection of these findings illustrates that Col1 and Col3 expression varies between samples by corresponding tumor heterogeneity, suggesting that inconsistent stromal cell content across the BRCA cohort confounds traditional expression analysis of collagen genes.

We hypothesized that mining for significant genomic correlations with Col1 and Col3 gene expression in the BRCA cohort should largely yield associations with stromal cell genes and extracellular matrix components subject to the same purity bias. In order to validate this, Pearson correlations and corresponding p-values were calculated independently for COL1A1, COL1A2, and COL3A1 versus the other 20,530 unique gene expression estimates from the RNA-seq platform for the TCGA BRCA cohort. Q-values were calculated by adjusting the p-values for false discovery rate using the Benjamini-Hochberg procedure. The resulting q-value matrices for each of COL1A1, COL1A2, and COL3A1 gene expression were ranked to obtain the top 200 gene hits, which were then input to g:Profiler for gene set and pathway enrichment analysis. The resulting number of significantly enriched gene sets in the top correlates for each of COL1A1, COL1A2, and COL3A1 were similar at 336, 286, and 297, with 243 of the gene sets overlapping between all 3 genes. Moreover, the most significantly enriched biological pathways within the top correlates for each of the Col1 and Col3 genes were identical extracellular matrix-based pathways (“extracellular matrix”,

GO:0031012; “extracellular matrix organization”, GO:0030198; “extracellular structure organization”, GO:0043062; and “collagen-containing extracellular matrix”, GO:0062023), demonstrating that expression analysis of Col1 and Col3 in tumors incidentally selects for similar associations that are subject to tumor purity biases.

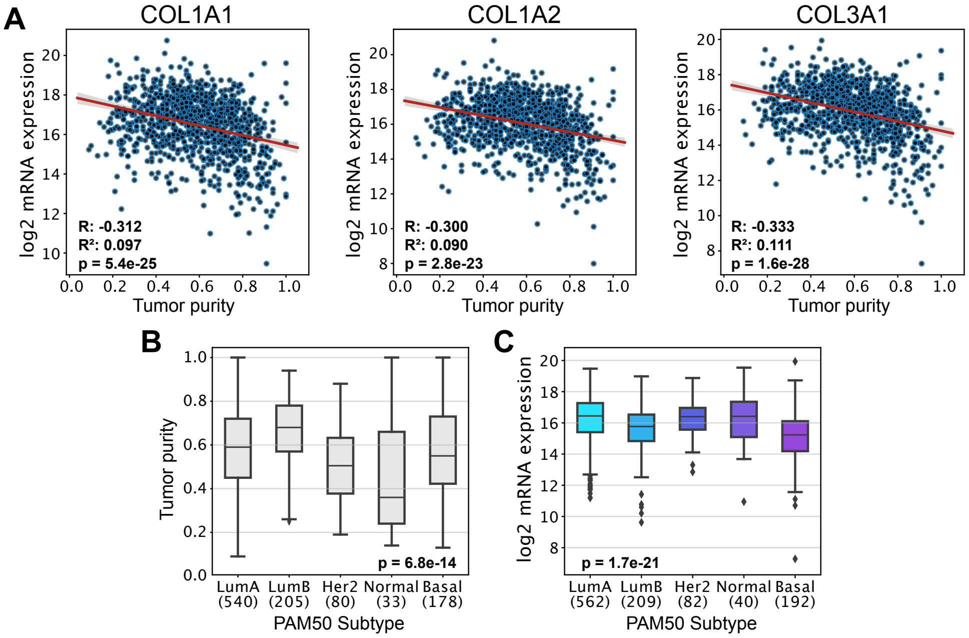

Figure S3: Sample heterogeneity skews expression analysis of bulk tumor tissue data. *(A) Gene expression of COL1A1, COL1A2, and COL3A1 inversely correlate with the estimated proportion of tumor cells within samples of the TCGA BRCA cohort (p<0.0001, Pearson correlation coefficient t-test), demonstrating that Col1 and Col3 expression within samples vary based on corresponding normal cell and tumor cell fractions. (B) Tumor purity values were found to significantly vary between PAM50 subtype groupings within the TCGA BRCA cohort (p<0.0001, Kruskal-Wallis H test). (C) COL1A1 and COL1A2 (not shown) and COL3A1 gene expression were found to significantly vary between PAM50 subtype groupings (p<0.0001, Kruskal-Wallis H test) in a pattern inverse to that of purity.*

To address the issue of varying stromal content between patient tumors and thereby allow us to evaluate the clinical implications of relative Col3 levels in patient tumors, we sought to analyze a ratio of Col1:Col3 gene expression. After calculating correlations between tumor purity and the ratios of COL1A1:COL3A1 and COL1A2:COL3A1 gene expression, we observed diminished relationships with corresponding p-values that, while still significant, were 15 to 22 orders of magnitude larger than those calculated between tumor purity and individual COL1A1, COL1A2, and COL3A1 gene expression (p<0.0001, Pearson correlation coefficient t-test) (Supp. Fig. S4A). Next, we again compared the distributions of the COL1A1:COL3A1 and COL1A2:COL3A1 expression ratios across PAM50 subtypes. Variation was notably

reduced between subtypes compared to the distributions of individual Col1 and Col3 gene expression, with only the COL1A2:COL3A1 ratio demonstrating significance (p<0.0001, Kruskal-Wallis H test) (Supp. Fig. S4B).

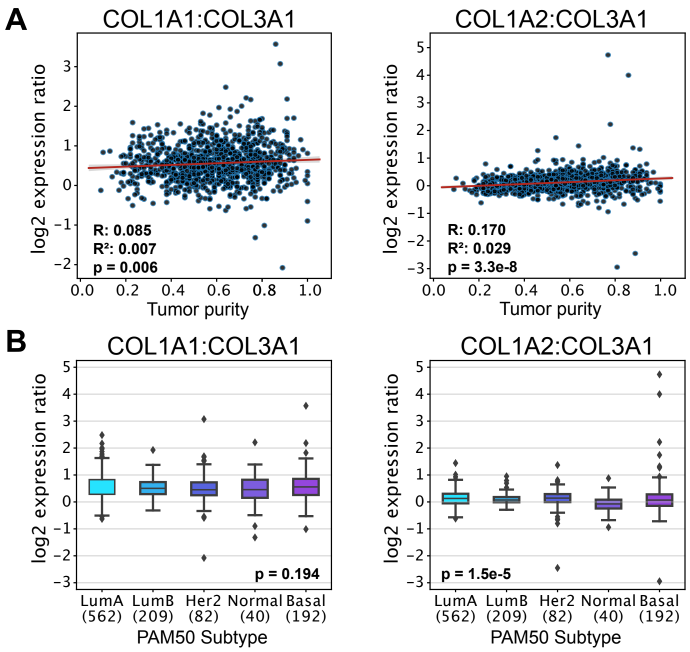

Figure S4: A biologically relevant Col1:Col3 expression ratio adjusts inter-tumor heterogeneity biases. *(A) (A) The ratios of the COL1A1:COL3A1 and COL1A2:COL3A1 gene expressions exhibit a visibly diminished relationship (Pearson correlation coefficient) with tumor purity compared to the relationship between individual gene expression and tumor purity in Supp. Fig. 2. (B) Comparing the distributions of the COL1A1:COL3A1 and COL1A2:COL3A1 expression ratios across PAM50 subtypes. The variation is notably reduced between subtypes, with only the COL1A2:COL3A1 ratio demonstrating significance (p<0.0001, Kruskal-Wallis H test).*

As validation of reduced tumor purity influence when examining the Col1:Col3 expression ratio, we repeated the genome-wide gene expression correlation analysis and identified the top 200 correlates between the COL1A1:COL3A1 and COL1A2:COL3A1 expression ratios for gene set and pathway enrichment assessment. While there were 286 to 336 significantly enriched gene sets within the top correlates for COL1A1, COL1A2, and COL3A1 alone, there were 831 significantly enriched gene sets within the top 200 gene expression correlates for the COL1A1:COL3A1 ratio, 8 significantly enriched gene sets for the COL1A2:COL3A1 ratio, and only 1 overlapping gene set between the two ratios ("nucleoplasm", GO:0005654). In addition, none of the most significantly enriched biological pathways for either ratio were extracellular matrix-related, suggesting that analysis of Col1:Col3 gene expression ratios demarcates Col1 and Col3 expression signals from stromal fraction bias.

Additional data from the Kaplan-Meier survival analysis of Col1:Col3 high and Col3:Col1 high patients groupings; COL1A1 high/low groupings; COL1A2 high/low groupings; and COL3A1 high/low groupings are provided below.

	Disease-free interval			Progression-free interval			Disease-specific survival			Overall survival		
	Col1:Col3 high	Col3:Col1 high	p-value	Col1:Col3 high	Col3:Col1 high	p-value	Col1:Col3 high	Col3:Col1 high	p-value	Col1:Col3 high	Col3:Col1 high	p-value
All BRCA	320	318	**0.029***	369	371	**0.037***	367	365	0.298	369	371	0.300
Luminal A	178	154	0.289	200	186	0.872	200	184	0.097	200	186	0.603
Luminal B	55	51	0.082	66	61	**0.015***	65	59	**0.038***	66	61	0.399
Her2	17	24	0.767	21	26	0.808	21	25	0.645	21	26	0.902
Normal	9	16	0.346	11	20	0.728	11	20	0.361	11	20	0.915
Basal	58	69	**0.036***	67	74	**0.031***	66	73	**0.026***	67	74	**0.011***

Figure S5: The Col1:Col3 expression ratio significantly associates with clinical outcome in TCGA BRCA. *Summary table with the number of patients classified as Col1:Col3 high and Col3:Col1 high that had available data for each clinical endpoint type and the accompanying p-values calculated from log rank tests comparing the Col1:Col3 high and Col3:Col1 high survival distributions. (*p<0.05).*

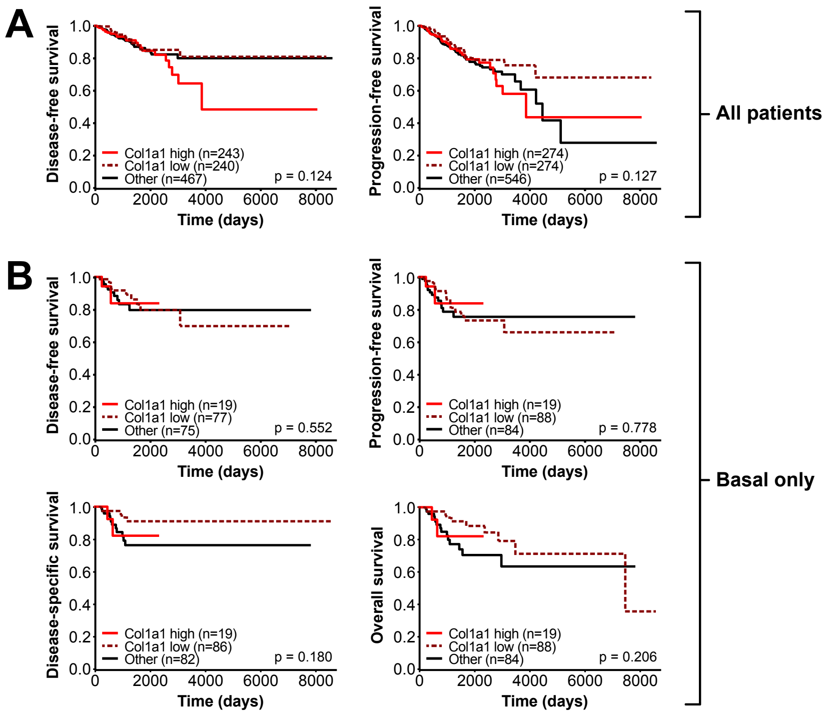

Figure S6: Survival curves comparing COL1A1 high and COL1A1 low patient groupings in TCGA BRCA.

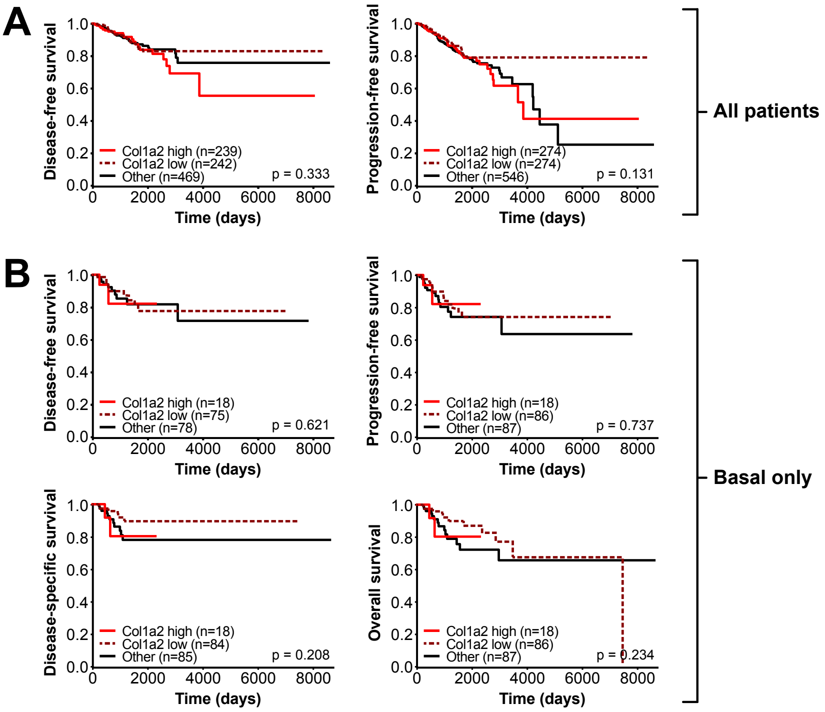

Figure S7: Survival curves comparing COL1A2 high and COL1A2 low patient groupings in TCGA BRCA.

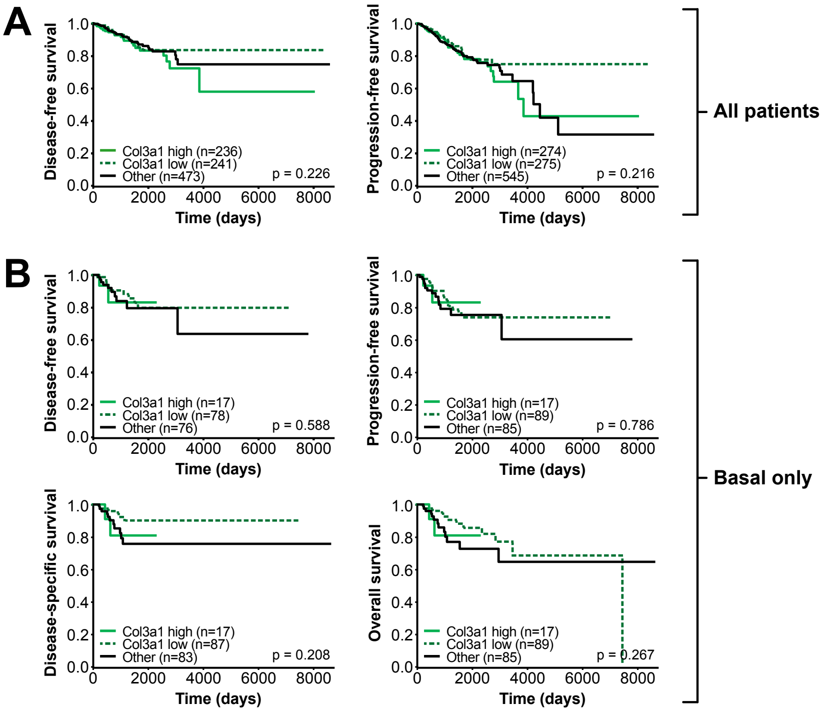

Figure S8: Survival curves comparing COL3A1 high and COL3A1 low patient groupings in TCGA BRCA.
